# Consistency of gene starts among *Burkholderia *genomes

**DOI:** 10.1186/1471-2164-12-125

**Published:** 2011-02-22

**Authors:** John Dunbar, Judith D Cohn, Michael E Wall

**Affiliations:** 1 Bioscience Division, Los Alamos National Laboratory, Los Alamos, NM, USA; 2 Computer, Computational, and Statistical Sciences Division, Los Alamos National Laboratory, Los Alamos, NM, USA; 3 Bioscience Division, Center for Non-Linear Studies, Computer, Computational, and Statistical Sciences Division, Los Alamos National Laboratory, Los Alamos, NM, USA

## Abstract

**Background:**

Evolutionary divergence in the position of the translational start site among orthologous genes can have significant functional impacts. Divergence can alter the translation rate, degradation rate, subcellular location, and function of the encoded proteins.

**Results:**

Existing Genbank gene maps for *Burkholderia *genomes suggest that extensive divergence has occurred--53% of ortholog sets based on Genbank gene maps had inconsistent gene start sites. However, most of these inconsistencies appear to be gene-calling errors. Evolutionary divergence was the most plausible explanation for only 17% of the ortholog sets. Correcting probable errors in the Genbank gene maps decreased the percentage of ortholog sets with inconsistent starts by 68%, increased the percentage of ortholog sets with extractable upstream intergenic regions by 32%, increased the sequence similarity of intergenic regions and predicted proteins, and increased the number of proteins with identifiable signal peptides.

**Conclusions:**

Our findings highlight an emerging problem in comparative genomics: single-digit percent errors in gene predictions can lead to double-digit percentages of inconsistent ortholog sets. The work demonstrates a simple approach to evaluate and improve the quality of gene maps.

## Background

Identification of gene boundaries--the first step in genome annotation--provides the foundation for subsequent comparative genomics. Unfortunately, errors occur. When gene-coding regions are identified, one of a multitude of possible translational start sites must be selected. Gene-finding algorithms such as Glimmer [1], Genemark [2] and Prodigal [3] score each possible start site based on multiple features (e.g. start codon identity and upstream ribosome binding site), but the highest scoring site is not always the true site used *in vivo*. For example Glimmer 3.02 [1], Genemark 2.6 [2] and Prodigal 1.20 [3] predict incorrect start sites for 9%, 5.5%, and 3.5% of 884 *Escherichia coli *genes with experimentally validated gene starts [3]. Genome-specific features such as %GC content can substantially reduce the performance of gene prediction algorithms [3]. Even when the accuracy per genome is high, the aggregation of errors among groups of genomes can produce a large fraction of flawed results and significantly undermine comparative analyses.

Erroneously chosen start sites have a dual impact. The N-terminus of the encoded peptide sequence and the length of the upstream intergenic region are both altered. These errors undermine subsequent informatics such as the similarity of orthologous genes and regulatory regions, predicted operon structure, and the prediction of regulatory motifs. In extreme cases, incorrect gene start sites abolish intergenic spaces, sometimes resulting in spurious gene overlaps. Nearly a thousand examples of spurious gene overlaps were documented in 338 bacterial genomes, of which *Burkholderia thailandensis *E264 was the worst case [4]. It is likely that a much greater number of less conspicuous errors exist. For example in a preliminary study, we noted that 47% of 116 pairs of orthologous transcription factors from *B. thailandensis *and *B. pseudomallei *had inconsistent start sites, despite the high average amino acid identity (93%) between the pairs. We also noted numerous inconsistencies in gene start sites for orthologous genes between three strains of *Burkholderia pseudomallei *despite the fact that the genome sequences were annotated by the same genome center and released within an 18-month period between 2005 and 2007. Thus, errors in gene start sites might be more common than suggested by spurious overlaps.

To assess the potential extent and impact of gene-calling errors, we performed a genome-wide analysis of orthologs across the *Burkholderia *genus. We focused on five species representing major clades within the genus. The most distantly related species have 97% 16S rRNA sequence similarity and a median amino acid identity of 71.4% between orthologous proteins. Potential gene-calling errors were identified by inconsistency in gene start positions among orthologs. Inconsistencies will, of course, represent a mixture of true biological variation and gene-calling errors. The two possibilities can be conclusively distinguished by genome-wide experimental validation [5-7]. However, since experimental validation is not currently practical for most genome sequences, conservative heuristics are needed that can parse inconsistencies into probable errors versus plausible evolutionary divergence. Toward this end, we examined the nature of inconsistencies in gene start sites among *Burkholderia *orthologs. Our findings suggest that probable errors can be distinguished. This distinction enables substantial improvements in gene maps, and identification of plausible biological variation with possible functional consequences.

## Results

### Consistency among orthologs

In the original Genbank records, the average number of genes per genome for the five species used in this study was 6999. From these, 2681 ortholog sets were identified containing a gene from each genome. DNA sequence alignments showed that only 47% of the sets had consistent (i.e. aligned) start sites. Given that this level of inconsistency might arise from gene-calling errors, we implemented a comparative genomics approach to assess whether consistency could be achieved. For this approach, we required a list of all possible start sites for each gene, generated in a consistent manner for the five target genomes. We also needed quality scores for each possible start site. The Prodigal gene-calling algorithm conveniently provided these data and had the added advantages of being self-tuning, easy to implement, and specifically designed for improved performance with GC-rich genomes such as the 68% GC *Burkholderia *genomes [3].

Prodigal predicted an average of 7026 genes per genome. A total of 2801 Prodigal ortholog sets were identified; 65% had consistent start sites. The same percentage was obtained when restricting the analysis to the 2659 ortholog sets containing equivalent Genbank and Prodigal genes. The substantial improvement in ortholog consistency arising simply from use of a different gene-calling algorithm illustrates the imperfection of gene calling and motivates the notion that inconsistent gene starts among closely related orthologs may often represent gene-calling errors.

**Figure 1:**
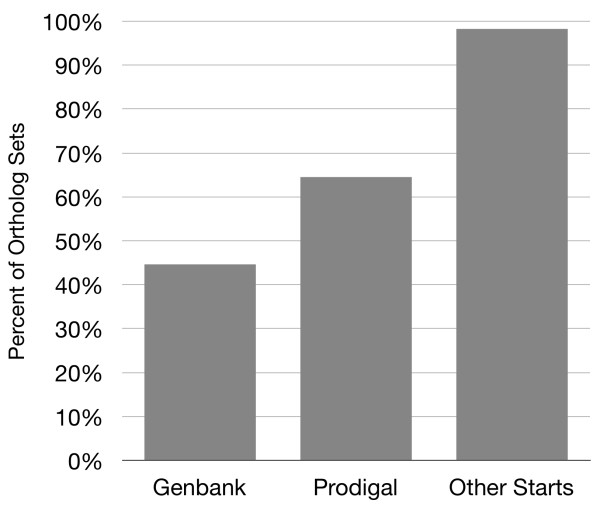
**Percentage of ortholog sets with consistent gene start sites based on Genbank gene maps, Prodigal gene maps, or Prodigal gene maps with revised start sites**.

To assess whether gene-calling errors could account for the 994 Prodigal ortholog sets with inconsistent starts (i.e. 35% of the 2801 sets; [Additional file 1]), we determined the maximum level of consistency theoretically achievable. For each ortholog set, we determined if a common start site existed in the multiple sequence alignment. If multiple common starts occurred, we selected the site with the highest average Prodigal quality score across the five genomes. With this approach, only 49 ortholog sets (1.7% of 2801 sets) lacked a common start. These sets represent either false orthologs or true evolutionary divergence among gene start sites. Consistent start sites were obtained for 98.3% of the 2801 ortholog sets (Figure 1). This increase over the Prodigal gene-calls involved modification of gene start sites for 3028 genes within 945 sets of orthologs. The 34% of Prodigal ortholog sets (945 of 2801) with correctable inconsistencies is only about 2-fold larger than the percentage expected if the Prodigal gene-calling error rate per genome was 3.5%, like *E. coli *[3], and the errors were uncorrelated among the genomes. Hence, all of the inconsistencies could conceivably be gene-calling errors.

For additional characterization, we sorted the 945 Prodigal ortholog sets with inconsistent starts into five groups according to the number of revisions (1 to 5) required to achieve consistency in each set. There were 292, 136, 43, 35, and 439 ortholog sets in the five groups respectively. For groups 1 to 4, inconsistent gene start sites occurred with nearly equal frequency among the five genomes, with the exception of *Burkholderia xenovorans*, which had about 2-fold higher occurrences in group 1 and 2-fold lower occurrences in groups 2 and 4 (Table 1). The distribution conflicts with the binomial distribution expected from random, uncorrelated gene-calling errors. In particular the sharp increase to 439 sets in group 5 suggests that the ortholog sets in this group are unlikely to represent random errors. Instead, many of these might be examples of true evolutionary divergence.

**Table 1:** Corrected gene starts by genome and number of corrections per ortholog set

	Revisions per ortholog set				
**Genome**	**1**	**2**	**3**	**4**	**5**

*B. ambifaria*	48	61	27	31	439
*B. pseudomallei*	47	63	22	34	439
*B. thailandensis*	38	60	26	29	439
*B. vietnamiensis*	52	55	30	31	439
*B. xenovorans*	107	33	24	15	439

Total revisions:	292	272	129	140	2195

To gain further insight, we revised the gene start sites for groups 1 to 5 to impose consistency, and then examined how the revisions altered the lengths, similarities, and subcellular locations of encoded proteins, as well as changes in the number and similarity of upstream intergenic regions as described below.

### Protein length

**Figure 2:**
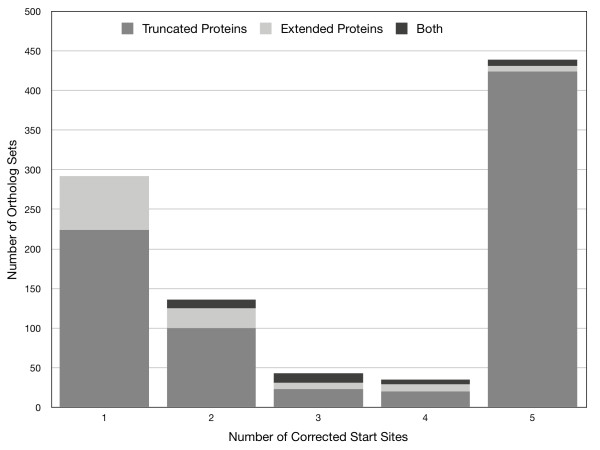
**Protein length impact of gene start site revisions in 945 ortholog sets**. The sets are grouped by the number of start-site revisions required to achieve consistency within each set. Shading indicates the type of protein alteration (i.e., truncation or extension) that occurred when start sites were revised to achieve consistency.

**Figure 3:**
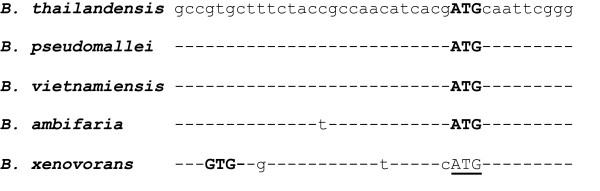
**Example of a corrected gene start site**. The alignment shows orthologous sequences from five *Burkholderia *genomes. Dashes indicate identical sequence relative to *B. thailandensis*. Bold text shows the Prodigal-predicted gene start sites. The *xenovorans *start site (bold) adds an 8 amino acid leader sequence to the encoded protein. Underlined text indicates the revised start site for *B. xenovorans*, imposed to achieve consistency.

**Figure 4:**
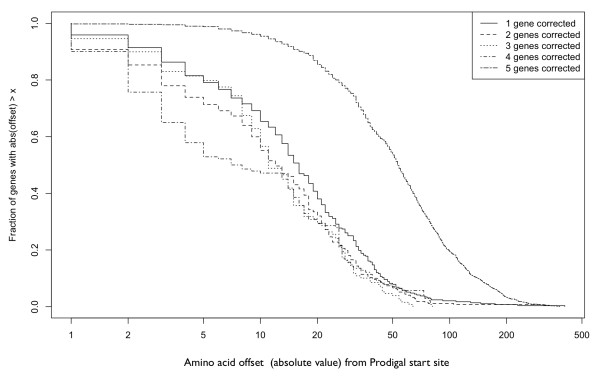
**Cumulative distribution of protein length changes in 945 ortholog sets requiring 1, 2, 3, 4, or 5 revisions to achieve gene start site consistency**.

Revision of gene start sites to obtain consistency within ortholog sets generally truncated the proteins (Figure 2). Figure 3 shows a typical example of a revised gene start in an ortholog set from group 1 (10.4% of all 2801 sets). The inconsistent start sites for group 1 typically arose from slight variations, often only a single base pair, in the upstream region containing the ribosome binding site, presumably altering the quality scores computed for competing start sites. For the group 1 ortholog sets, revision of gene start sites lengthened 68 proteins by 30 amino acids on average (median = 16), whereas in the remaining 224 cases the encoded protein was truncated by 22 amino acids on average (median = 16) (Figure 4). Similarly, in groups 2, 3, and 4 a minor fraction of proteins was lengthened, on average by 16, 13, and 13 amino acids, respectively. The majority of revised start sites caused average protein truncations of 20, 18, and 18 amino acids. Group 5, which accounted for 15.7% of the 2801 total ortholog sets, was unusual. A small fraction of the proteins were lengthened, on average by 29 amino acids. Most proteins were truncated. The fraction of truncated proteins was much higher than the other 4 groups (98% versus 63 to 78%). The average length of the truncation was also higher: 69 amino acids (median = 54), more than 3-fold larger than the other groups (Figure 3). These results show exceptional divergence in gene start sites for ortholog sets in group 5. This supports the contention that gene-calling error is the most parsimonious explanation for the inconsistencies in ortholog sets, except those sets in group 5, which are more likely to represent true divergence.

### Ortholog similarity

**Figure 5:**
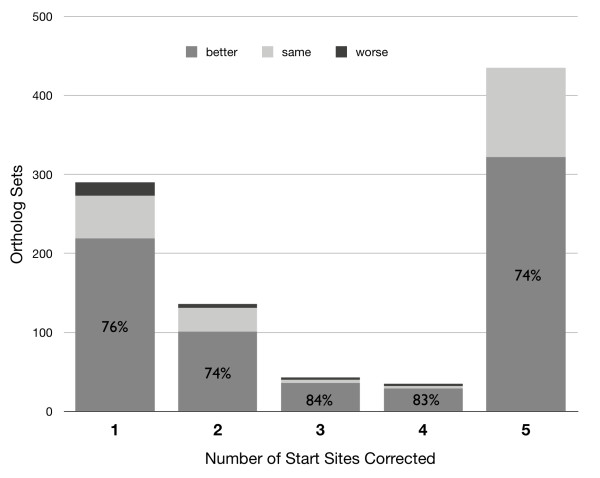
**Impacts of gene start site revisions on the calculated sequence identity of orthologous proteins**. Impacts are summarized as better (higher protein identity), same, or worse (lower protein identity). The changes are relative to protein similarities computed prior to revision (i.e. using the default Prodigal start sites).

**Figure 6:**
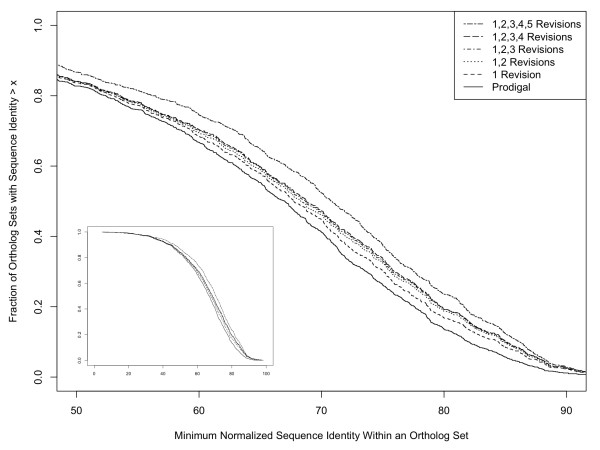
**Cumulative distribution of orthologous protein sequence identity values**. The minimum sequence identity values from 945 Prodigal ortholog sets were plotted, illustrating the impact of gene start site revisions over the range of observed protein conservation. Main plot shows majority of data, between 50 and 90% protein sequence identity, while inset shows entire range of values.

Revision of gene start sites generally improved the similarity of orthologs, as expected (Figure 5). Reducing length differences should increase the calculated similarity. Gene corrections that trim noncoding (upstream) DNA sequence from the gene can also increase protein similarities because noncoding DNA tends to be more variable than true coding sequence. For each ortholog set, the lowest of the ten pair-wise identity values in the set was used to indicate the maximum extent of protein divergence. The median for all Genbank ortholog sets was 71.4% identity. For the 1807 Prodigal ortholog sets where the Prodigal-predicted starts sites aligned without any corrections, the identity values ranged from 7 to 100% with a median of 77.8%. The median values for Prodigal ortholog sets in the five groups with inconsistent start sites ranged from 63.5% to 71.4% before corrections and increased 3-5% after corrections were made (Table 2, Figure 6). The net impact of applying Prodigal and our comparative genomics approach to refine *Burkholderia *gene maps boosted the median sequence identity for ortholog sets from 71.4% to 74.5%.

**Table 2:** Protein sequence identity for ortholog sets before and after modification of gene start sites

	Revisions per ortholog set					
	**0**	**1**	**2**	**3**	**4**	**5**

**Before Corrections**						
Maximum	100.0	97.3	92.1	93.0	84.3	92.5
Median	77.8	69.8	70.2	71.4	67.1	63.5
Minimum	6.8	17.1	25.6	32.5	19.4	4.8
**After Corrections**						
Maximum		98.2	97.2	95.8	87.3	94.6
Median		73.9	73.4	75.9	71.2	67.5
Minimum		17.1	33.7	32.0	19.0	6.7

### Subcellular location

**Figure 7:**
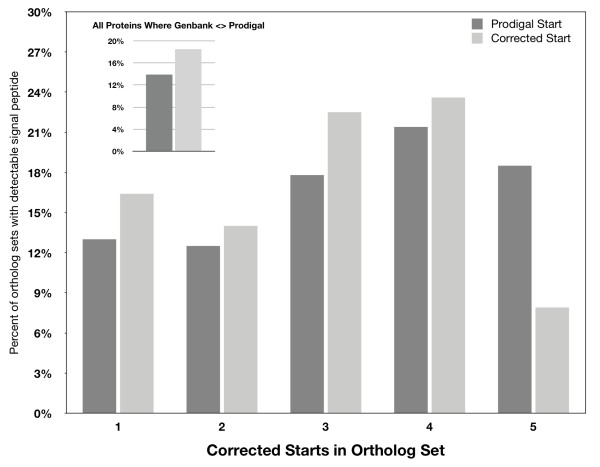
**Impact of gene start site revisions on signal peptide detection for proteins in 945 Prodigal ortholog sets with inconsistent gene starts**. PSORTb was used to detect signal peptides for proteins with Prodigal start sites or with corrected start sites to achieve consistency. Inset: comparison of signal peptide detection among equivalent Genbank and Prodigal proteins for which start site predictions varied.

**Figure 8:**
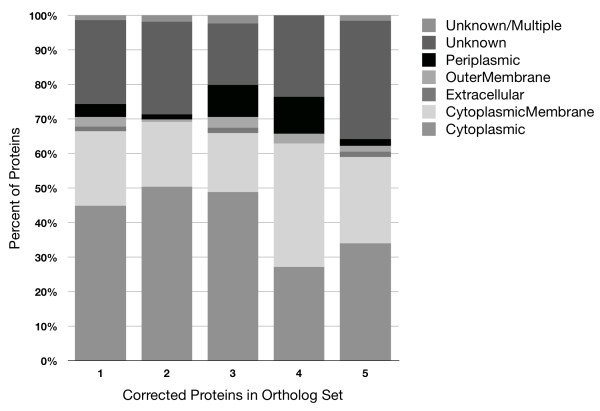
**Predicted subcellular localization of proteins in 945 Prodigal ortholog sets with revised gene starts**.

Proteins secreted to the periplasm or the extracellular medium typically have N-terminal signal peptides that direct protein trafficking. If inconsistent gene start sites arise from gene-calling error, revisions should increase the number proteins containing detectable signal peptides. Among Genbank predicted genes, 15.4% had a detectable signal peptide. However, for the subset of genes where the Prodigal start site differed from the original Genbank start site, the percentage of proteins with detectable signal peptides was 13.9% using the Genbank start sites and 18.5% using the Prodigal start sites. Thus, revision of Genbank start sites for this set provided a 33% improvement in signal peptide detection. For the 945 Prodigal ortholog sets with inconsistent start sites, revising the starts sites improved detection of signal peptides by 26, 12, 26, and 10% for proteins in groups 1 to 4 (Figure 7). Detection of signal peptides was dramatically reduced by 57% for proteins in group 5 with revised gene start sites. This provides further evidence that gene-calling error may account for most of the inconsistencies in groups 1 to 4, whereas true evolutionary divergence is the most plausible factor for group 5. For groups 1 to 5, the distribution of proteins among subcellular locations varied (Figure 8), but the significance of the variation is unknown.

### Intergenic regions

Eliminating probable gene-calling errors in the Genbank gene maps increased the number of ortholog sets for which a complete set of upstream IGRs could be obtained. A complete set was defined as a set containing five IGR sequences, one from every genome. Inconsistent gene start sites sometimes abolish an intergenic region in one or more genomes, yielding incomplete sets. For the Genbank ortholog sets, 70.0% had complete sets of upstream IGRs, whereas 83.9% (2351 of 2801) of the Prodigal ortholog sets had extractable IGRs. Additional revisions for the 945 Prodigal orthologs with inconsistent start sites added only 19 complete IGR sets (adopting corrections in groups 1 to 4), boosting the total to 84.6%. If group 5 revisions were included, 106 additional IGR sets would occur. In total, correcting probable gene start-site errors in the Genbank gene maps yielded a 21% improvement in the fraction of ortholog sets with extractable upstream IGRs. Thus, refining gene maps can have a dramatic impact on regulatory genomics, which depends on the capacity to extract and compare IGRs upstream of orthologous genes.

**Figure 9:**
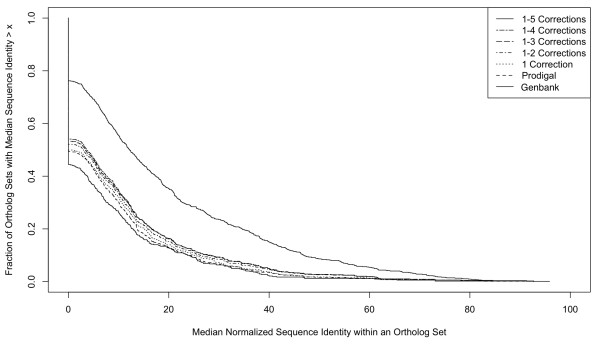
**Cumulative fraction plot of median IGR sequence identity for incremental corrections to ortholog sets with correctable start sites**.

Improved consistency of gene start sites also increased the sequence similarity of upstream IGRs (Figure 9). Because IGRs are more variable than coding sequences, we used the median sequence identity among all pairwise IGR comparisons within an ortholog set. We restricted this analysis to ortholog sets with a complete set of IGRs greater than 10 bp in length because BLAST similarity scores for shorter sequences could not be computed. The overall median of the identity values for IGRs upstream of ortholog sets with correctable start sites increased from 11.9% identity (Genbank) to 13.4 (Prodigal) to 14.2 (group 1) to 14.7 (groups 1-2) to 14.8 (groups 1-3) to 15.0 (groups 1-4) to 22.1 (groups 1-5). An increase in IGR sequence identity can arise from reductions in length differences and from misincorporation of coding sequence in the IGR. Inclusion of coding sequence presumably accounts for the substantial increase in IGR sequence similarity associated with revision of ortholog sets from group 5. This observation provides a further indication that the inconsistent gene starts among orthologs in group 5 more likely represent true divergence, not gene calling-error.

If inconsistent gene start sites reflect true divergence rather than gene calling error, evolutionary divergence might occur more broadly in the local gene neighborhood. To explore this, we determined if the adjacent gene upstream of ortholog sets in groups 1 to 5 was consistent (i.e. orthologous) across genomes. Consistency indicates conserved gene blocks. Inconsistencies imply recombination events that alter the gene neighborhood. This analysis did not reveal any significant trend. About 29% of the 1807 Prodigal ortholog sets with aligned starts had inconsistent upstream genes. For groups 1 to 5, the percentage of ortholog sets with a non-conserved gene neighborhood ranged from 21 to 44%. The only notable finding was that 71% of the 49 ortholog sets that had no common start sites showed divergence in the upstream gene neighborhood, substantially higher than the percentage for all other groups of ortholog sets. A qualitatively similar outcome was obtained when using upstream and downstream genes jointly to define conserved gene blocks, instead of only the upstream gene.

#### GC skew

For twenty ortholog sets sampled from groups 1 to 5, we manually assessed whether the revised gene start sites were compatible with the beginning of a protein-coding sequence as indicated by GC skew profiles [8,9]. In organisms with high GC content, the wobble position in coding sequence tends to exhibit an elevated GC bias [8]. This bias can reveal potential coding sequences and correct reading frames [8]. In ideal cases, the GC skew clearly increases as coding sequence begins, which can indicate the general area where a gene start site is likely to occur. Owing to lack of automation, we examined only the first twenty ortholog sets in [Additional file 1]. The sets included the first twenty orthologous genes from *B. thailandensis *chromosome I (Bth_I0003 to Bth_I0088). For the eight sets representing groups 1 to 4, the revised gene start sites were as good or better than the original gene start sites in terms of compatibility with the GC skew profile (data not shown). In contrast, revised start sites for the twelve ortholog sets from group 5 appeared to interrupt protein-coding sequence (data not shown). These results provide further evidence that the ortholog sets in group 5 represent true evolutionary divergence in the location of gene start sites.

## Discussion

Small error rates in gene calling can have a large aggregate effect on comparative genomics. For five species spanning the genus *Burkholderia*, we found Genbank records contained substantial inconsistencies in gene start sites for predicted orthologs, most of which appeared to represent gene-calling error. *Burkholderia thailandensis, B. pseudomallei, B. ambifaria, B. vietnamiensis*, and *B. xenovorans *had 2681 genes in common. Of these ortholog sets, about 53% had inconsistent gene start sites (Figure 1). The percentage was reduced to 35% simply by using Prodigal, a different gene-finding algorithm designed for better performance with GC-rich genomes like the 68% GC *Burkholderia *genomes [3]. Using comparative genomics, the inconsistency of gene starts among orthologs could be credibly reduced to 17 to 25%. Our findings illustrate the aggregate impact of probable gene-calling errors and demonstrate a facile approach to distinguish probable error from evolutionary divergence.

To correct probable errors in the Genbank gene maps, we combined the use of a different gene-calling algorithm and a comparative genomics approach. Sole use of comparative genomics is possible. However, a list of alternative start sites and their quality scores provides a quantitative foundation for choosing between possible common start sites. The Prodigal software conveniently provides a list of alternative starts and their quality scores. Fortuitously, Prodigal also made substantial improvements to the *Burkholderia *gene maps, reducing the number of revisions that would otherwise have been made by comparative genomics alone. Uniform application of any gene finding algorithm to a set of genome sequences seems likely to improve consistency. However, substantial differences in the performance of gene-calling algorithms do exist. For example, the average error rates (i.e. incorrect gene start site predictions) for Glimmer3 and Prodigal with GC-rich genomes are 9.3 and 3.9%, respectively [3]. Our findings are consistent with the reported error rates.

The 53% inconsistency rate we observed for *Burkholderia *ortholog sets from the original Genbank gene maps implies an intrinsic gene-calling error rate of about 14% (= 1-(1-.53)^1/5^)) per *Burkholderia *genome. This rate is close to the 13.1% Glimmer3 error rate reported for the 68% GC-rich *Halobacterium salinarum *genome [3]. A similar calculation using the 35% inconsistency among ortholog sets from Prodigal gene maps for the five *Burkholderia *species yields a Prodigal error rate of 8.3% per *Burkholderia *genome. The reported error rate for Prodigal with GC-rich genomes (greater than 65% GC) ranges from 3.1 to 6.4%. Thus, a substantial fraction of the 945 Prodigal ortholog sets with inconsistent gene starts is likely to represent gene-calling error. It is not surprising that gene finding algorithms make errors. At present, identification of gene start sites includes a probabilistic component. Because gene start sites do not have a definitive sequence signature, true start sites will sometimes be obscured by noise. This underscores the value of a comparative genomics approach for post-processing predicted start sites. A noisy signal in one genome can be counterbalanced by clearer signals in other genomes.

Revision of probable gene start site errors tended to shorten the genes. Sequence alignments (e.g., Figure 2) routinely showed that the truncated "leader" sequence of a revised gene was not a unique insert, but instead corresponded to the upstream intergenic region of the other orthologs. If the original inconsistent start sites were correct, it would imply a complicated evolutionary scenario: conversion of upstream intergenic sequence into coding sequence, in many cases subsuming promoters and transcription factor binding sites and possibly requiring evolution of new regulatory elements further upstream. The most appealing null model for closely related species is the simplest one--divergence of orthologous genes is minimal, unless compelling evidence suggests otherwise. Improving the consistency of gene start sites satisfies this null model. Our revisions also improved the calculated similarity of the upstream intergenic regions, encoded proteins sequences, and the detection of signal peptides. As these observations are not truly orthogonal, experimental validation is ultimately needed to confirm that the revised gene start sites are the correct sites *in vivo*.

**Figure 10:**
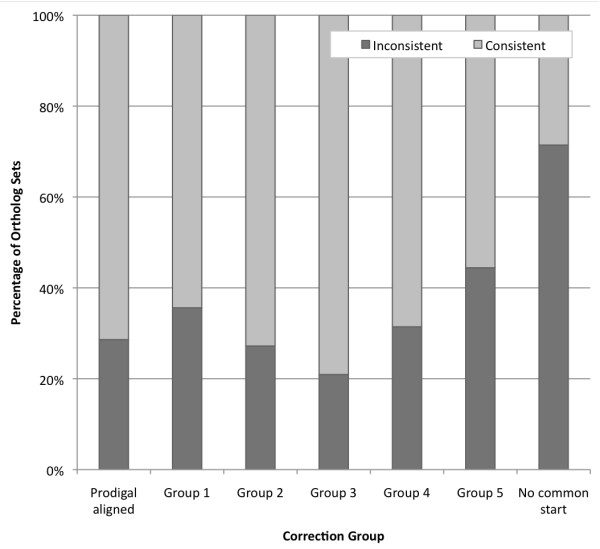
**Consistency of adjacent upstream gene for ortholog sets in different gene start-site correction groups**. The upstream gene context for an ortholog set was defined as consistent if all five upstream genes (one from each genome) belonged to a single ortholog set.

Two groups of ortholog sets appeared to represent true evolutionary divergence. One group was the 49 ortholog sets for which no common start sites existed. The 43% median amino acid identity for proteins in this group was exceptionally low. Only a third of the ortholog sets in this group occurred in a conserved gene block (Figure 10), This group represents either true divergence or false orthologs. The other candidate group, group 5, contained ortholog sets in which all five genes in each set had to be revised to achieve consistent gene starts. This group contained 439 sets, about 16% of the total number of Prodigal ortholog sets for the five genomes. This group was unusual in several ways. First, the number of ortholog sets was 10-fold higher than the two preceding groups. Second, gene start site revisions typically required exceptionally large truncations of the genes. Third, revisions substantially *decreased *detection of signal peptides whereas there was an increase for the other groups. Fourth, revisions appeared to interrupt protein-coding sequence as indicated by GC-skew profiles [8,9]. It is unlikely that the bulk of orthologs in this group were false orthologs. Half the ortholog sets in this group had protein similarities greater than 63%. We excluded all obvious paralogs from the analysis, and over half of the 439 ortholog sets represented conserved gene blocks. Thus, even if some paralogs remained in the group, they did not account for most of the sets. Combined, these observations suggest evolutionary divergence is the most plausible explanation for the inconsistencies in this group.

Real evolutionary divergence of gene starts can have important functional consequences. Gene expression may change if altered gene start sites require evolution of new upstream transcriptional regulatory motifs. Protein translation rates may change if the -4 to +37 region around the new start site has a different mRNA folding propensity [10]. Changing the N-terminus of the encoded protein can also alter the rate of protein degradation [11] or the protein's subcellular location [12]. It is reasonable to expect that evolutionary divergence of gene start sites would occur in some metabolic functional categories more than others. Thus, for ortholog sets in group 5--the group most likely representing evolutionary divergence--one might expect enrichment of specific COG categories. However, we found no statistically significant enrichment of COG categories in group 5 or in groups 1 to 4. The lack of enrichment of COG categories showed that neither gene-calling error nor evolutionary divergence appeared biased toward a particular type of gene or annotation.

## Conclusions

It is important to distinguish evolutionary divergence from gene-calling errors. Gene-calling errors substantially degrade the integrity of comparative genomics, which increasingly serves as a foundation for biology and medicine. Our results demonstrate that plausible cases of evolutionary divergence can be distinguished from probable gene calling errors. This simple approach facilitates analyses of functional consequences and changes in cellular behavior that may arise from true divergence. Expansion of this comparative genomics approach can significantly improve gene maps.

## Methods

### Genomes

Completed genomes were used for the following five species spanning the genus *Burkholderia*: *B. thailandensis *E264, *B. pseudomallei *1710b, *B. ambifaria *MC40-6, *B. vietnamiensis *G4, and *B. xenovorans *LB400. For each organism, the default gene maps from NCBI RefSeq were obtained in October 2008 from the following files: NC_007650.ptt, NC_007651.ptt, NC_007434.ptt, NC_007435.ptt, NC_010551.ptt, NC_010552.ptt, NC_010557.ptt, NC_009254.ptt, NC_009255.ptt, NC_009256.ptt, NC_007951.ptt, NC_007952.ptt, NC_007953.ptt.

#### Orthologs

An all-versus-all BLASTP identity matrix was constructed using the following normalized sequence identity metric: (BLAST percent identity)*(BLAST alignment length)/max(Query_length, Target_length). An ortholog set was defined as the set of pan-reciprocal best BLASTP matches containing a single gene from each of the five genomes. Thus, ortholog sets containing paralogs from a genome with a tie for best match were excluded from further analysis. The standard default E-value cutoff of 10 was used for BLASTP matches. The worst BLASTP E-values (ranging from 8.5 to 10^-5^) occurred with 32 orthologs sets representing very short proteins.

### Prodigal gene predictions

New gene predictions for each genome were obtained using the Prodigal bacterial gene prediction software [3], version 1.10. In addition to the selected start and stop sites for each gene, we obtained from Prodigal the list of all potential start positions and their respective quality scores. Most genes were identical to the pre-computed Prodigal gene predictions available through Genbank; however, 5% were different. We attribute the differences to two factors: (1) the version of Prodigal used by Genbank (1.20 rather than 1.10), and (2) the way Prodigal was run. In our case, for each strain we ran a separate Prodigal "training" step using a concatenation of the fasta files for all chromosomes to ensure consistent gene calling across the entire genome.

### Equivalent Genes

The default genes predicted for each genome in Genbank and Prodigal-predicted genes were deemed equivalent if they shared the same stop position.

### Sequence Alignment

For each ortholog set, a multiple sequence alignment was performed using MUSCLE [13] version 3.7 on extended DNA sequences, beginning 250 bases upstream of the first potential start position and finishing at the end of the coding sequence.

### Selection of new gene starts

Positions within the alignment where all genomes shared a possible start position were identified. If all five Prodigal-selected start sites were aligned, no change was made to any of the gene start sites. Otherwise, shared start positions within the alignment were ranked by the average (across genomes) Prodigal quality score for each start site. The highest-ranked aligned start site was then chosen as the new start site for the set of orthologs.

### Comparison of Intergenic Regions (IGR)

Intergenic sequences were extracted from genomic sequence based on either the original Genbank gene maps, the Prodigal gene maps, or the Prodigal gene maps amended with our revised start sites. Sets of synonymous IGRs were obtained as follows: for each set of orthologous genes, the upstream IGR from each genome was extracted if the length of the IGR in each of the five genomes was greater than 10 nucleotides. Pairwise comparisons of IGRs in each set were performed using BLASTN with a default cutoff of E = 10. Results were used to obtain the normalized sequence identity measure (described above) for the IGRs. If BLASTN did not return a score for a pair of IGR sequences, the normalized sequence identity for the pair was set to 0. We calculated the median of the normalized sequence identity scores for all pairs in an ortholog set as a single metric of IGR sequence identity within the set. To distinguish IGR variation arising from sequence divergence versus recombination events, we assessed the conservation of the upstream flanking gene. If the upstream gene in all five genomes belonged to the same ortholog set, the local gene context was recorded as "conserved".

### Signal Peptide Detection and subcellular localization

The presence or absence of signal peptides and subcellular localization was determined using PSORTb v3.0.2 [14].

### Data Management and Codebase

All data was managed and stored within an Oracle 11 g database. The data management and analysis pipeline were written in a combination of Java, PL/SQL and C-shell scripts.

## Authors' contributions

JD and MEW designed and directed the work and performed some of data analysis. JDC performed most of the analysis. JD wrote the manuscript with contributions from JDC and MEW. All authors read and approved the final manuscript.

## Supplementary Material

Additional file 1 **994 Prodigal ortholog sets with inconsistent start sites**. The Excel file provides information about the 994 ortholog sets with inconsistent start sites, including the genes within each set and the gene start site revisions required to achieve consistency within each set. Click here for file
